# Cluster-Based Improved Isolation Forest

**DOI:** 10.3390/e24050611

**Published:** 2022-04-27

**Authors:** Chen Shao, Xusheng Du, Jiong Yu, Jiaying Chen

**Affiliations:** School of Information Science and Engineering, Xinjiang University, Urumqi 830046, China; shaochen@stu.xju.edu.cn (C.S.); yujiong@xju.edu.cn (J.Y.); chenjiaying@stu.xju.edu.cn (J.C.)

**Keywords:** Isolation Forest, clustering, *k*-means, selection matrix

## Abstract

Outlier detection is an important research direction in the field of data mining. Aiming at the problem of unstable detection results and low efficiency caused by randomly dividing features of the data set in the Isolation Forest algorithm in outlier detection, an algorithm CIIF (Cluster-based Improved Isolation Forest) that combines clustering and Isolation Forest is proposed. CIIF first uses the *k*-means method to cluster the data set, selects a specific cluster to construct a selection matrix based on the results of the clustering, and implements the selection mechanism of the algorithm through the selection matrix; then builds multiple isolation trees. Finally, the outliers are calculated according to the average search length of each sample in different isolation trees, and the Top-n objects with the highest outlier scores are regarded as outliers. Through comparative experiments with six algorithms in eleven real data sets, the results show that the CIIF algorithm has better performance. Compared to the Isolation Forest algorithm, the average AUC (Area under the Curve of ROC) value of our proposed CIIF algorithm is improved by 7%.

## 1. Introduction

Outlier detection is an important research direction in the field of data mining, which aims to uncover the unusual data present in a dataset [1,2]. The most widespread definition of an outlier is that proposed by Hawkins [3]. Outliers are those data objects that deviate from most of the data set, raising the suspicion that these deviations are not generated by random factors, but by a completely different mechanism. The main reasons for outliers are anomalies in the data itself and errors caused by the collection of data.

Isolation Forest is an unsupervised detection method specially designed based on the isolation of outliers [4]. The method isolates outliers by splitting the data space through a random hyper plane, reflecting the characteristic that outliers are easily isolated. With high accuracy and low computational complexity, this method is widely used in the industry. However, the Isolation Forest uses a completely random selection of features and feature values when constructing isolation trees, and the overly random selection leads to a possible invalid selection of feature values, resulting in divided features as interference features and affecting the detection results.

In response to the limitations of the Isolation Forest method, this paper proposes an algorithm CIIF that combines clustering and Isolation Forest. First, the proposed method clusters the dataset using the *k*-means method [5] and constructs a selection matrix based on the results of the clustering. Then, the process of isolation trees construction splits the sample set using a selection matrix, which can effectively avoid the error caused by the defects of the traditional Isolation Forest. Finally, outliers are calculated based on the average search length of each sample in each decision tree, and the n samples with the highest outliers are listed as outliers.

Our main contributions are summarized as follows:

1. The proposed method introduces a pre-selection mechanism to improve the shortcomings of Isolation Forest which are the unstable detection results and the low efficiency caused by randomly dividing features of the dataset.
2. The proposed method uses the *k*-means algorithm to obtain the distribution of the dataset, which is used to construct a selection matrix to implement a pre-selection mechanism.
3. The proposed method introduces the parameter selection degree *I* to control the influence of the pre-selection mechanism on the method and avoid overfitting.

The methods for outlier detection can be classified into distribution-based methods, nearest-neighbor-based methods, clustering-based methods, neural network-based methods, classification-based methods, and isolation-based methods.

The distribution-based outlier detection algorithm is one of the first proposed algorithms, whose main idea is to assume that the data distribution of a dataset fits a statistical model and define outliers as those points that are in the low probability region [6]. Classic representative models include the Gaussian distribution model [7,8,9,10], etc.

The outlier detection algorithm based on nearest neighbors is to detect outliers based on the relationship between all data and their nearest neighbors. This class of methods can be divided into two categories: distance-based methods [11,12,13] and density-based methods [14,15,16,17]. Classic representative algorithms are the KNN (K-Nearest-Neighbor) algorithm [18] based on distance and the LOF (Local Outlier Factor) algorithm based on density [19].

The clustering-based outlier detection algorithm [20] is an unsupervised algorithm whose main idea is to detect outliers by analyzing the relationship between data points and clusters, which has good results for most data sets [21,22,23]. The disadvantage of the clustering-based outlier detection algorithm is that the main purpose of the algorithm is to obtain the distribution characteristics of the dataset, and the detection efficiency for outlier points is not optimal, and the model needs to be adjusted according to the actual application, so it cannot be flexibly applied to different datasets. The DBSCAN (Density-Based Spatial Clustering of Applications with Noise) algorithm is the representative of this type of method [24,25,26].

The classification-based outlier detection algorithm trains a classifier from a labeled dataset and uses this classifier to detect outliers [27]. The algorithm also has a disadvantage. When the amount of data in the training dataset is insufficient, the efficiency and accuracy of the trained classifier will fall as expected.

With the development of deep learning techniques, neural network-based methods [28,29,30] have also advanced. This type of method has high detection accuracy and good performance on different types of datasets. However, the models for this type of approach are usually more complex and require a lot of time to train the model. Some of the popular methods are as follows: Autoencoder Ensemble [31,32,33], GAN (Generative Adversarial Network)-based model [34,35,36], Graph neural network [37,38,39,40], etc.

The isolation-based outlier detection method defines data that can easily be isolated as outliers [41,42,43]. Isolation Forest is the representative of this type of method, which constructs multiple isolation trees by splitting the sample space through hyper planes. These isolation trees are completely random in the selection of attributes and split values each time during the construction process. These isolation trees constitute the Isolation Forest. The Isolation Forest algorithm defines those points that are easily isolated as outliers, which tend to be the leaf nodes closest to the root node in the isolation trees. These outliers are too different from other samples in the sample space and are far from the distribution center of the sample. Therefore, we can locate potential outliers by calculating the average finding length of sample points in the entire forest.

## 2. Materials and Methods

The Isolation Forest algorithm can cause the constructed isolation tree to fail to accurately reflect the difference between normal and outlier points due to the random selection of split values in the process of constructing the isolation tree, which finally affects the detection results.

**Figure 1:**
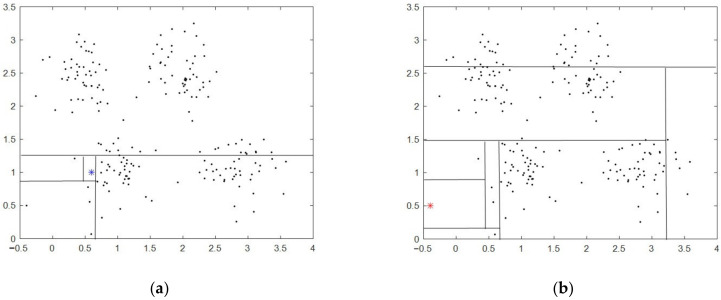
The principle of IF, (**a**) normal data; (**b**) outlier. The blue asterisk indicates normal data, and the red asterisk indicates an outlier.

As shown in Figure 1, the blue asterisk indicates normal data, and the red asterisk indicates an outlier, due to the random selection of the split value, normal data may be more likely to be isolated than an outlier.

**Figure 2:**
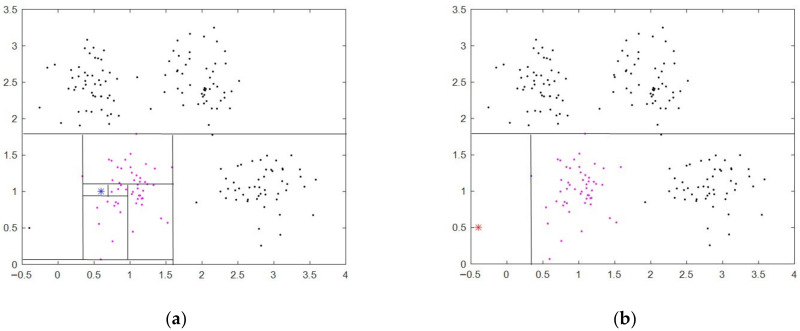
The principle of CIIF, (**a**) normal data; (**b**) outlier. The blue asterisk indicates normal data and the red asterisk indicates an outlier, the data in the selection cluster are indicated by the purple dots.

To improve the shortcomings of the IF, CIIF introduces a pre-selection mechanism. The main idea is to select a suitable cluster based on the data distribution of the dataset, and preferentially select the boundary and center of that cluster as the split values, which is shown in Figure 2:

As shown in Figure 2, the outliers are isolated more accurately in CIIF.

CIIF is divided into two phases, the training phase, and the evaluation phase. The training phase is divided into two steps, firstly, the construction of the selection matrix, and then the construction of the Isolation Forest.

### 2.1. Training Phase

#### 2.1.1. Selection Matrix

The CIIF algorithm is an unsupervised outlier detection algorithm that analyzes the distribution of the dataset through the *k*-means clustering algorithm, divides the dataset into *k* clusters, and selects the appropriate cluster as the selection cluster *Cs*.

**Definition** **1.** *selection cluster* *C_s_*

*Let the dataset X be divided into k clusters C_1_, C_2_, …, C_k_ by the k-means clustering method, and each cluster is scored as follows:*

(1) $$Score\left(c_{i}\right)=\frac{\sum_{j=1}^{m_{i}}dist\left(c_{i}x_{j}\right)}{n_{i}}$$

*where dist(c_i_,x_j_) is the Euclidean distance, n_1_, n_2_, … n_k_ are the amount of data contained in each cluster, and c_1_, c_2_, … c_k_ are the cluster centers of each cluster.*

Define the cluster with the lowest score as the selection cluster *C_s_*.

The choice of selection cluster *C_s_* directly affects the performance of the whole algorithm. Different clusters as the selection cluster will lead to a large difference in the results of the algorithm. Clusters with large data-to-data differences as the selection cluster can seriously degrade the performance of the algorithm, and clusters with larger amounts of data are more suitable as the selection cluster than those with smaller amounts of data. Therefore, it is necessary to score each cluster to determine the best choice of selection cluster.

The selection matrix *S* is built based on the selection cluster.

**Definition** **2.** *selection matrix* *S*

*Let the dimension of the dataset be d, and define the selection matrix as:*

(2) $$S=\left\{S_{1},S_{2},\cdots S_{d}\right\}$$

Define the maximum value of the selection cluster *C_s_* in dimension *d* as *Max*(*c_d_*), the minimum value as *Min*(*c_d_*), and the average value as *Mean*(*c_d_*), then:

(3) $$s_{d}=\{Max(c),Min(c),Mean(c)\}$$

The construction process of matrix *S* is shown in Algorithm 1.


**Algorithm 1.**
*Get-S(D, k)*
** Input**: *D*-input data, *k*-number of clusters ** Output**: selection matrix *S***Initialize***S**C* ← *k*-means(*D*, *k*)//Cluster the dataset, return the result *C* = {*C*_1_,*C*_2_,…,*C_k_*}**for***i* = 1 to *k* **do**  *score_i_* ← *score_i_*∪*Score*(*C_i_*);//Score each cluster**end for***s* ← argmin(*score_i_*)//Get the serial number of the selection clustern ← size(*C_s_*);//*C_s_* = {*x*_1_,*x*_2_,…,*x_n_*}, *x_n_* = {*n_n_*_1_,*n_n_*_2_,…,*n_nd_*}, *d*-number of dimensions/* Construct the selection matrix S*/**for***I* = 1 to *d* **do**  *Sam* ← *Φ*  **for** *j* = 1 to *n* **do**    *Sam* ← Sam∪*n_ij_*  **end for**  *s_i_* ← {*Min*(*Sam*), *Max*(*Sam*), *Mean*(*Sam*)}  *S* ← *S*∪*s_i_*;**end for****return***S*

The selection matrix *S* is the set of data boundaries and means of the selection cluster *C_s_* in each attribute, which reflects the distribution characteristics of the selection cluster. The CIIF algorithm implements the selection mechanism for split value selected by the selection matrix when constructing the forest. In this process, the selection of the split values will be prioritized from the optional points of the selection matrix *S* in that attribute.

#### 2.1.2. Isolation Forest

Set the selection degree *I* to control the degree of influence of the selection matrix *S* on the algorithm.

**Definition** **3.** *selection degree I*

*The selection degree I is defined as the maximum number of times that split value can be selected by the selection matrix S in each attribute.*

The degree of selection is a parameter that controls the randomness of the algorithm and is determined artificially. The larger the value of *I*, the more the forest is influenced by the selection cluster *C_s_* and the lower the randomness; the smaller the value of *I*, the less the forest is influenced by the selection cluster *C_s_*, the greater the randomness, and the closer it is to the original Isolation Forest algorithm; when the selection degree *I* is 0, the algorithm is the original Isolation Forest algorithm at this time.

**Definition** **4.** *discriminant matrix J*

*Define the discriminant matrix J as the record of the number of split values decided by each dimension according to the selection matrix S during the construction of the Isolation Forest by CIIF:*

(4) J(d)=i

*where d represents the dimension and i is the record value. Equation (4) indicates that CIIF performs i times split value selection for d dimensions in constructing the Isolation Forest.*

The isolation tree is the core of the whole CIIF algorithm. To construct the isolation tree, we first select a subsample from the sample space, use the subsample as the root node of the isolation tree, then randomly select an attribute, choose a value from the candidate values of the selection matrix *S* in the range of the subsample in the selected attribute, use the value as the split value, and update the record of the selected attribute in the discriminant matrix; If the candidate values of the selection matrix in the selected attribute are not in the range of the subsample or the record of the selected attribute in the discriminant matrix *J* is greater than the selection degree *I*, then randomly select a value as the split value in the range of the subsample.

The subsample space is divided into two subspaces according to the split value, and the data with value less than the split value in the selected attribute are grouped in the left subspace, and the data with value greater than the split value are grouped in the right subspace, and the two subspaces are the two subtrees of the root node. The above process is repeated recursively for both subtrees until the leaf nodes contain only one data, or all the data in the leaf nodes have the same value; or the height of the tree exceeds the limit, at which point the isolation tree construction is completed. The construction process of the isolation tree is shown in Algorithm 2.
**Algorithm 2.** *iTree(D, l, L, S, J)* **Input**: *D*-input data*, l*-current tree height*, L*-height limit*, S*-selection matrix*, J*-discriminant matrix  **Output**: an *iTree*get selection degree *I***if***l > L***or***|x| <=* 1 **then**  **return** *exNode***else**  let *Q* be a list of attributes in *D*  randomly select an attribute *q*∈*Q*  let *k* be the serial number of *q* in *D*  *s* ← {*x* | *x* ⋲ *S*(*k*, : ),*min* <= *x* <= *max*}  **if** *s* ≠ *Φ* and *J*(*k*) < *I* **then**    randomly select a split point *p* from *s*    *J*(*k*) ← *J*(*k*) + 1  **else**
    randomly select a split point *p* from *max* and *min* values of attribute *q* in *D*  **end if**  *Dl* ← *filter*(*D*, *q* < *p)*  *Dr* ← *filter*(*D*, *q* > =*p)*  **return** *inNode*{*Left* ← *iTree(Dl*, *l +* 1, *L*, *S*, *J*),    *Right* ← *iTree*(*Dr*, *l +* 1, *L*, *S*, *J*),    *SplitAtt* ← *q,*    *SplitValue* ← *p*}**end if**

Construct multiple isolation trees to form an isolation forest, the construction process of an isolation forest is shown in Algorithm 3.
**Algorithm 3.***iForest(D, t, X)* **Input**: *D*-input data, *t*-number of isolation trees, *X*-subsampling size ** Output**: a set of *iTrees***Initialize***Forest***Initialize***J*set height limit *L* = *ceiling*(log_2_*X*)*S* ← *Get-S*(*D*, *k*) //get the selection matrix**for***I* = 1 to *t* **do**  *D’* ← *Sample*(*D*,*X*)  *Forest* ← *Forest*∪*iTree*(*D’*, 0, *L*, *S*, *J*)**end for****return***Forest*

### 2.2. Evaluation Phase

After the training phase, the proposed method will calculate the outlier scores of all data points in the isolation forest with the following outlier score calculation formula:

(5) $$score(x,n)=2^{-\frac{E(h(x))}{c(n)}}$$

where *h*(*x*) is the path length of sample *x* from the root node to the leaf node where it is located, *E*(*h*(*x*)) is the expectation of path length *h*(*x*) in an isolated forest, and *c*(*n*) is the average of the path lengths of all data points, calculated as follows:

(6) $$c(n)=2H(n-1)-\frac{2(n-1)}{n}$$

where *H*(*i*) is the Harmonic series, which can be calculated as ln(i) + γ, and γ is the Euler’s constant, which is approximately equal to 0.5772156649.

When *E*(*h*(*x*)) tends to 0, the outlier score tends to 1, and the data point *x* is judged to be an outlier. On the contrary, if the score tends to 0, the data point *x* will be judged as a normal point. When the score tends to 0.5, it is not possible to determine whether the data point *x* is an outlier.

This algorithm has two stages, the first stage is to construct the selection matrix and the second stage is the improved Isolation Forest algorithm. The first stage clusters the data set by the *k*-means algorithm, and the selection matrix is constructed according to the clustering results. The computational complexity of computing the Euclidean distance of the data set is O(n^2^), the computational complexity of the *k*-means algorithm is O(n), and the computational complexity of constructing the selection matrix is O(n), so the computational complexity of the first stage is O(n^2^). The second stage is the improved Isolation Forest algorithm with linear computational complexity. Thus, the computational complexity of the whole improved algorithm is O(n^2^).

## 3. Results

### 3.1. Subsection

To verify the effectiveness of the algorithm, experiments were conducted on 11 different publicly available real datasets from UCI and ODDS [44,45,46,47,48,49,50,51,52,53,54], and the AUC value was used as the Accuracy Metric of the algorithm.

The specific attributes of datasets are shown in Table 1. The breastw dataset is the Wisconsin breast cancer diagnosis dataset, which is a high-dimensional dataset publicly available at UCI and contains diagnostic data for malignant and benign tumors. The diagnostic data for malignant tumors are labeled as outliers. The annthyroid dataset is a thyroid disease dataset, which is divided into two categories: noisy and normal, and the noisy data are labeled as outliers. The arrhythmia dataset, which is a cardiac arrhythmia dataset, divides the data into multiple categories, the eight categories with less data are labeled as outliers. The pima dataset is an Indian diabetes dataset, divided into two categories: abnormal and normal; the abnormal data are labeled as outliers. The vertebral dataset is a genomic dataset with six dimensions, classifying data into normal and abnormal categories, the abnormal data are labeled as outliers. The wine dataset is a dataset of results of chemical analyses of wines made from three different grapes from the same region of Italy, which identified the number of 13 components contained in the three wines, the data for one of the wines are labeled as outliers. The ionosphere dataset is a binary dataset with 34 dimensions, classifying the data into bad and good classes, removing an invalid attribute, the bad class data are labeled as outliers. The shuttle dataset is the flight data of the aircraft; the data are divided into two categories, the data of the category with smaller number are labeled as outliers. The cardio dataset is the fetal heart rate measurements on the ECG that have been processed by a professional physician. The data are divided into three categories: normal, suspicious, and pathological, with the suspicious category discarded and the pathological category labeled as outliers.

### 3.2. Evaluation Metric

For a binary classification algorithm, data samples can be classified into four categories based on the classification results and true labels: True Positive (TP), False Positive (FP), True Negative (TN), and False Negative (FN), as shown in Table 2.

Area under the Curve of ROC (AUC) is the value of the area between the Receiver Operating Characteristic (ROC) curve and the horizontal coordinate. The ROC curve is a curve on a two-dimensional plane with the horizontal coordinate of the false-positive rate (FPR) and the vertical coordinate of the true-positive rate (TPR). The formula for calculating FPR and TPR is as follows:

(7) $$\mathrm{TPR}=\frac{\mathrm{TP}}{\mathrm{TP}+\mathrm{FT}}$$

(8) $$\mathrm{FPR}=\frac{\mathrm{FP}}{\mathrm{TN}+\mathrm{FP}}$$

AUC is calculated as:

(9) $$\mathrm{AUC}=\frac{\sum_{i_{p}}rank_{i_{p}}-\frac{M(M+1)}{2}}{MN}$$

where *i_p_* denotes a positive sample, *rank* is the sample serial number, *M* is the number of positive samples, and *N* is the number of negative samples. the AUC value is generally between 1 and 0.5, and the closer the AUC value is to 1, the better the performance of the algorithm. If the AUC value is below 0.5, the algorithm is not applicable to the detection dataset.

### 3.3. Experimental Results

Six typical outlier detection algorithms are used as comparison algorithms with the proposed CIIF to compare the AUC values and computational times on 11 datasets. The six comparison algorithms are Isolation Forest, LOF, KNN, COF (Connectivity-based Outlier Factor) [55], FastABOD (Fast Angle-Based Outlier Detection) [56], and LDOF (Local Distance-based Outlier Factor) [57].

Table 3 shows the AUC values of each algorithm on the 11 datasets and highlights the best AUC value with the second-highest AUC value on each dataset. By comparing the AUC values of each algorithm, we can see that the CIIF performs well on the annthyroid, arrhythmia, speech, vertebral, wine, and ionosphere datasets, and has a significant improvement compared to the Isolation Forest and other comparison algorithms; outperforms other comparative algorithms on thyroid and shuttle dataset, with less difference compared to the Isolation Forest; outperforms the original Isolation Forest algorithm and other comparison algorithms on the breastw dataset, and differs less from the COF; outperforms the Isolation Forest and other comparison algorithms on the pima dataset, and differs less from FastABOD. The performance on the cardio dataset is slightly worse than the original Isolation Forest algorithm and COF algorithm, and less different from the KNN algorithm.

**Figure 3:**
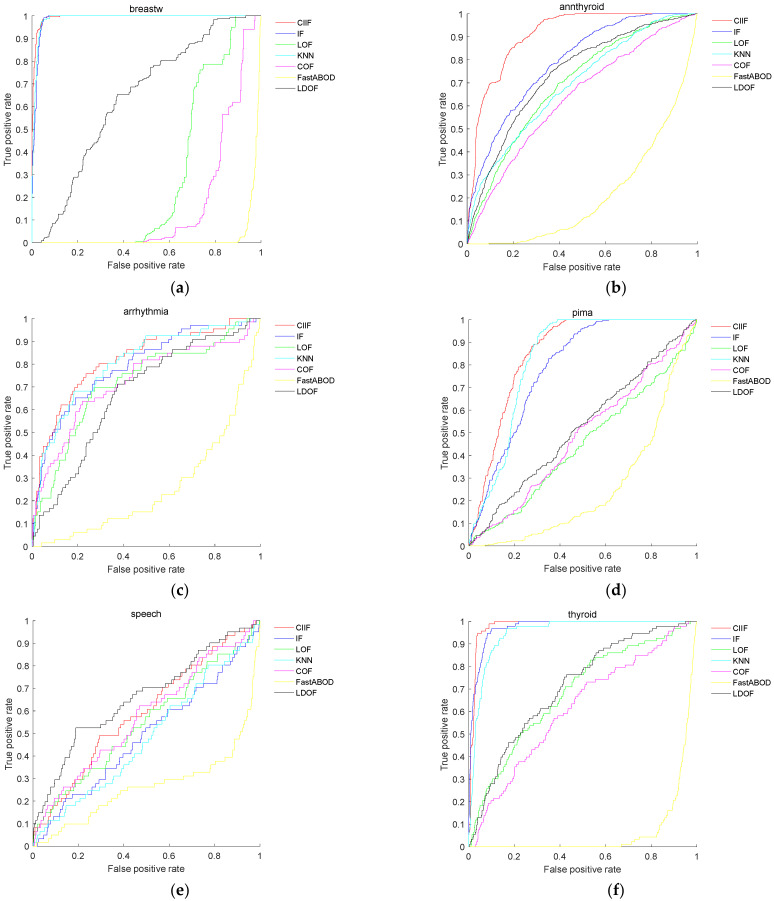

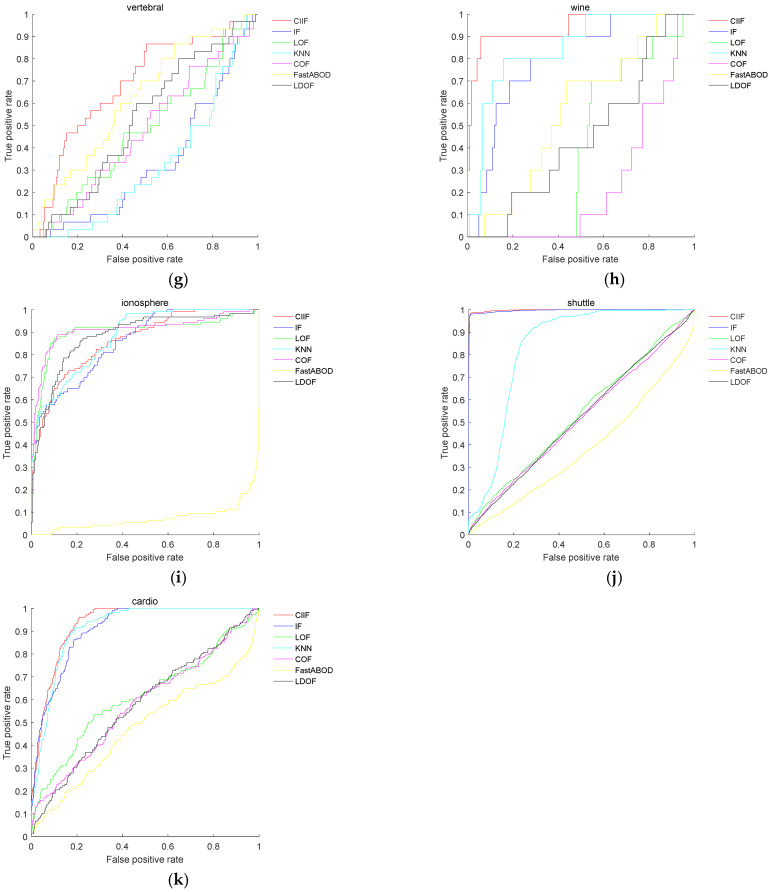
ROC curve of several algorithms in several datasets: (**a**) breast; (**b**) annthyroid; (**c**) arrhythmia; (**d**) pima; (**e**) speech; (**f**) thyroid; (**g**) vertebral; (**h**) wine; (**i**) ionosphere; (**j**) shuttle; (**k**) cardio.

Figure 3 shows the ROC curves of CIIF and other comparison algorithms on 11 datasets. The ROC curves of the proposed algorithm on the annthyroid, pima, thyroid, vertebral, wine, and cardio datasets are above the other algorithms; The ROC curves of the proposed algorithm on the shuttle, breast, and pima datasets nearly overlap with those of the Isolation Forest and are higher than those of other algorithms. On the speech dataset, CIIF does not work as well as LDOF. On the ionosphere dataset, CIIF does not work as well as LDOF, KNN and LOF. The results of comparing six state-of-the-art algorithms on eleven real-world datasets show that CIIF achieves the highest Area under ROC Curve (AUC) on nine datasets. Thus, the CIIF outperforms the IF and the other comparison algorithms in overall performance.

**Figure 4:**
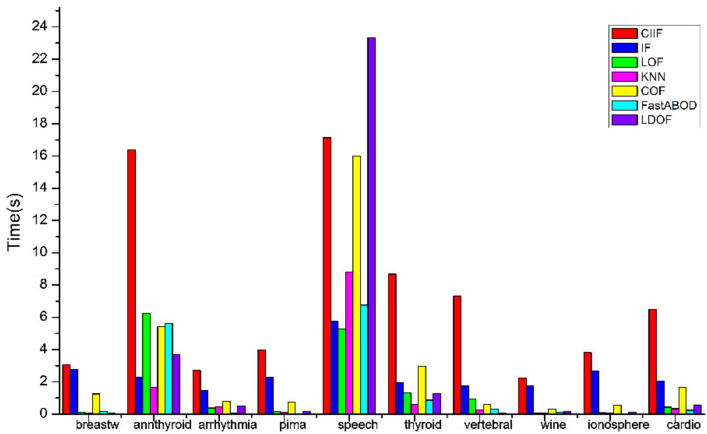
Computational times(s).

**Figure 5:**
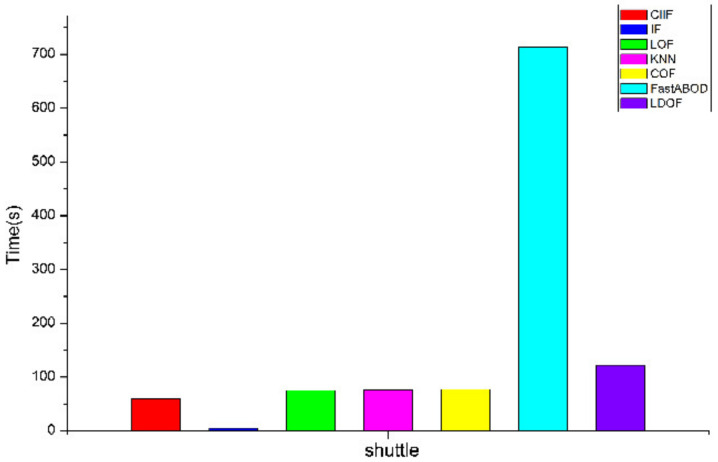
Computational time of shuttle(s).

As shown in Figure 4, the proposed algorithm has a higher computational time than LOF, KNN, COF, FastABOD, LDOF on datasets with smaller datasets. From Figure 5, the difference between the computational time of LOF, KNN, COF, LDOF, and the CIIF is not significant on the datasets with larger data volume such as shuttle data set, and the computational time of FastABOD is even much higher than the proposed algorithm. The computational time of the Isolation Forest is smaller than that of the CIIF on each dataset, but the CIIF has higher AUC values and better detection results on most of the datasets.

The experimental results show that CIIF has good detection performance on most of the datasets; the computational time on the datasets with less data is slightly higher than other algorithms, but within the acceptable range; the computational time on the datasets with larger data is smaller or not much different compared to other algorithms. Therefore, the CIIF is effective and feasible.

### 3.4. Parameter Analysis

The effect of selection degree *I*, number of subsampling *X*, on the proposed algorithm was analyzed experimentally on annthyroid, arrhythmia, pima, ionosphere, shuttle, and cardio dataset.

#### 3.4.1. Effect of Selectivity *I*

**Figure 6:**
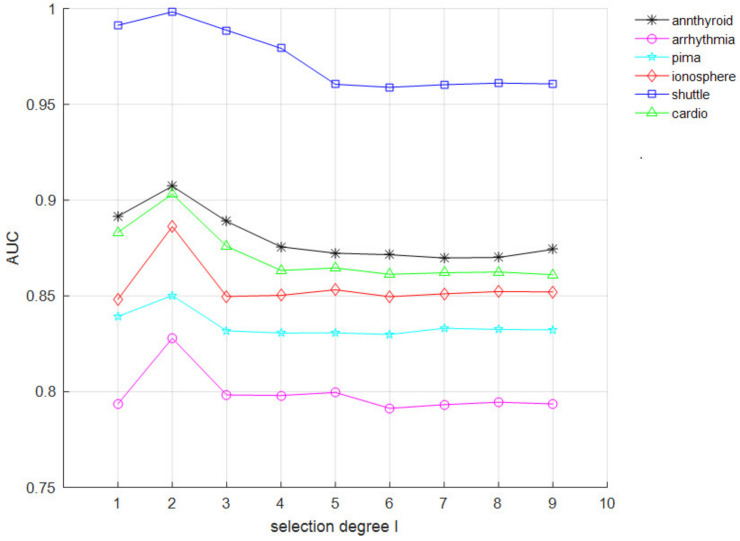
AUC impact of *I*.

Experiments were conducted with different *I* on six datasets. The range of *I* was set to integers from 1 to 10, because the proposed algorithm is no different from the Isolation Forest when *I* is less than 1, and the AUC values tend to be smooth when *I* is greater than 10. The experimental results are shown in Figure 6.

From the results, the influence of different *I* on each dataset is small. When *I* = 2, the AUC values reach the optimal value on each dataset and then decrease. Therefore, in the CIIF, the value of the selection degree *I* is generally set to 2, so that the CIIF can achieve the optimal performance on most dataset.

#### 3.4.2. Effect of Selectivity Subsampling X

Experiments were conducted on the datasets with large data such as annthyroid, speech, and shuttle, to explore the effect of subsampling *X* on the results. Because the subsampling number is too large for datasets with smaller data, they will no longer be subsampled, but use all the data to construct isolation trees, which eventually leads to all isolation trees in the isolation forest being constructed from the same set of samples.

**Figure 7:**
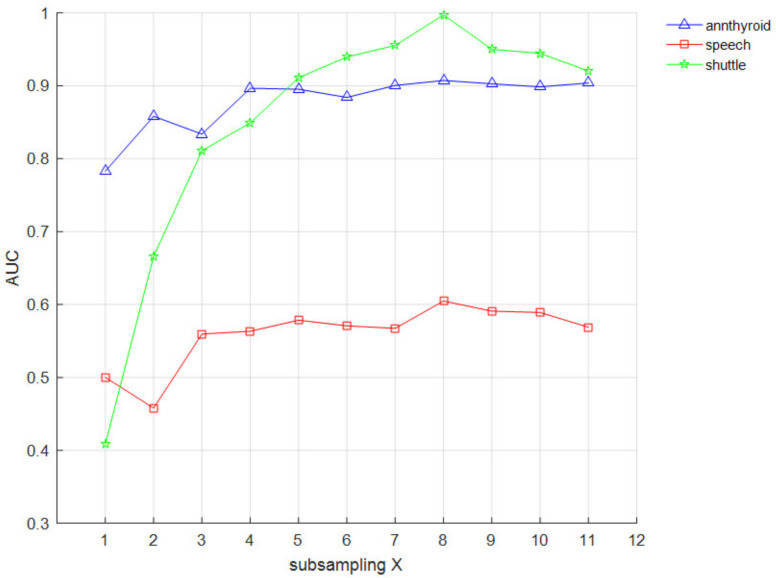
AUC impact of subsampling *X*.

From Figure 7, the detection performance of the CIIF on the shuttle dataset increases with the subsampling *X* and reaches the best at subsampling of 256. The detection performance on the annthyroid and speech datasets starts to level off at subsampling of 256, before which there are large fluctuations in detection performance. When the subsampling is too small, the detection performance of the CIIF is poor and unstable; when the subsampling is too large, the sample set will contain too many normal samples, leading to a certain degradation in performance and leading to greater time cost. Therefore, the best comprehensive performance of the proposed algorithm is achieved when the subsampling is 256.

IF takes a completely random selection of attributes and split values in the training process, ignoring the distribution characteristics of the dataset itself, so, as a result, the constructed isolation forest cannot accurately reflect the isolation of each sample, resulting in a decrease in detection accuracy. CIIF takes account of the distribution characteristics of the dataset and performs a heuristic training process based on these characteristics, resulting in better performance.

## 4. Conclusions

In this paper, we propose an improved isolation forest algorithm, which constructs a selection matrix to realize the pre-selection mechanism of attribute values for isolation forest division by clustering and analyzing the data distribution of the dataset, avoiding the problem of low accuracy caused by too much randomness of the Isolation Forest. Experiments on 11 datasets on UCI and ODDS verified the effectiveness of the algorithm. In the experiments, it was found that the performance of the *k*-means algorithm is too greatly affected by the given *k* value, and there is no less lossy way to select the appropriate *k* value for a data set, and the result of the clustering algorithm directly affects the performance of the CIIF, so the next step is to study the effect of different clustering algorithms on the CIIF and the improvement of *k*-means algorithm.

## Figures and Tables

**Table 1:** Testing dataset.

Dataset	Data Volume	Dimension	Number of Outliers	Outlier Ratio %
breastw	683	9	239	34.9927
annthyroid	7200	6	534	7.4167
arrhythmia	452	274	66	14.6018
pima	768	9	268	34.8958
speech	3686	400	61	1.6549
thyroid	3772	6	93	2.4655
vertebral	240	6	30	12.5
wine	129	13	10	7.7519
ionosphere	351	33	126	35.8974
shuttle	49,097	9	3511	7.1511
cardio	1822	21	176	9.6122

**Table 2:** Confusion matrix of classification results.

Actual	Forecast	
	Positive	Negative
Positive	TP	FN
Negative	FP	TN

**Table 3:** AUC results on 11 real-world datasets AUC of several algorithms. The highlighted data indicate the best and second best values on each data set.

Data	AUC						
	CIIF	IF	LOF	KNN	COF	FastABOD	LDOF
breastw	**0.9922**	0.9876	0.2421	**0.9881**	0.1273	0.6220	0.6394
annthyroid	**0.9073**	**0.8212**	0.6958	0.6938	0.6523	0.2153	0.7377
arrhythmia	**0.8392**	**0.8035**	0.5092	0.5092	0.7229	0.2562	0.5092
pima	**0.8502**	0.8064	0.4491	**0.8275**	0.4859	0.2580	0.5221
speech	**0.6048**	0.4539	0.5467	0.4821	0.5747	0.2662	**0.6592**
thyroid	**0.9799**	**0.9749**	0.6836	0.9481	0.6121	0.1642	0.7098
vertebral	**0.6911**	0.3659	0.4846	0.3238	0.4805	**0.6270**	0.5281
wine	**0.9403**	0.7829	0.4008	**0.8462**	0.2319	0.5647	0.4496
ionosphere	0.8624	0.8527	0.8643	**0.8793**	0.8529	0.1738	**0.8831**
shuttle	**0.9983**	**0.9968**	0.5184	0.6339	0.5534	0.4172	0.5208
cardio	**0.9305**	0.9042	0.6128	**0.9161**	0.5796	0.4759	0.5798
